# Filling Teeth after the Lining Membrane Has Become Exposed

**Published:** 1850-10

**Authors:** 


*Filling Teeth after the Lining Membrane has become Exposed.*—Dr. W. W. Codman, of Boston, informed us, while on a visit to that city last summer, that he had been in the habit, for several years, of filling teeth, under certain circumstances, after the lining membrane had become exposed, and with very great success. He also stated, that he was of the opinion, from a number of experiments which he had made, that the pulp of the tooth, when the operation is successful, sooner or later ossified. He presented us with several teeth in which this had actually occurred.

In a former number of the Journal, we took occasion to offer a few remarks on filling teeth under such circumstances; and in another place have treated upon the subject at some length. Still we do not feel fully warranted in recommending it very confidently. We design, nevertheless, to give the result of our experience and observations upon the subject, in a future number of the Journal, and in the meantime we would be glad if those members of the profession who have been in the habit of performing the operation, would furnish us with such facts in relation to it, as may have come to their knowledge.

